# ROCK inhibition reduces morphological and functional damage to rod synapses after retinal injury

**DOI:** 10.1038/s41598-020-80267-4

**Published:** 2021-01-12

**Authors:** Éva Halász, Marco A. Zarbin, Amy L. Davidow, Laura J. Frishman, Peter Gombkoto, Ellen Townes-Anderson

**Affiliations:** 1 Department of Pharmacology, Physiology and Neuroscience, Rutgers New Jersey Medical School, 185 South Orange Avenue, Newark, NJ 07103 USA; 2 Institute of Ophthalmology and Visual Science, Rutgers New Jersey Medical School, 90 Bergen Street, Newark, NJ 07103 USA; 3 Department of Biostatistics and Epidemiology, Rutgers School of Public Health, 683 Hoes Lane West, Piscataway, NJ 08854 USA; 4 College of Optometry, University of Houston, 3507 Cullen Blvd, Houston, TX 77204 USA; 5 Center for Molecular and Behavioral Neuroscience, Rutgers University, 197 University Avenue, Newark, NJ 07102 USA

**Keywords:** Biochemistry, Cell biology, Drug discovery, Molecular biology, Neuroscience, Physiology, Structural biology, Anatomy, Diseases, Medical research

## Abstract

Retinal detachment (RD) causes damage, including disjunction, of the rod photoreceptor-bipolar synapse, which disrupts vision and may contribute to the poor visual recovery observed after retinal reattachment surgery. We created a model of iatrogenic RD in adult female pigs to study damage to the rod-bipolar synapse after injury and the ability of a highly specific Rho-kinase (ROCK) inhibitor to preserve synaptic structure and function. This model mimics procedures used in humans when viral vectors or cells are injected subretinally for treatment of retinal disease. Synaptic disjunction by retraction of rod spherules, quantified by image analysis of confocal sections, was present 2 h after detachment and remained 2 days later even though the retina had spontaneously reattached by then. Moreover, spherule retraction occurred in attached retina 1–2 cms from detached retina. Synaptic damage was significantly reduced by ROCK inhibition in detached retina whether injected subretinally or intravitreally. Dark-adapted full-field electroretinograms were recorded in reattached retinas to assess rod-specific function. Reduction in synaptic injury correlated with increases in rod-driven responses in drug-treated eyes. Thus, ROCK inhibition helps prevent synaptic damage and improves functional outcomes after retinal injury and may be a useful adjunctive treatment in iatrogenic RD and other retinal degenerative diseases.

## Introduction

Retinal detachment (RD), the separation of the neural retina from the underlying retinal pigment epithelium (RPE), is a well-known cause of visual loss and has a major impact on quality of life^1^. Although the retina can be reattached by various surgical procedures, the final visual outcome is often unsatisfactory^2–8^.

The reasons for poor visual recovery are not completely understood. Recognized factors include the duration, extent, and height of RD as well as involvement of the macula, which contains the cone-rich fovea^2,3,5,7,9,10^. Another factor may be injury-induced rearrangement of neural circuits in the retina. First described by Erickson et al. in 1983^11^, rod presynaptic terminals retract from the outer plexiform layer (OPL) after RD, resulting in disjunction of the first synapse in the visual pathway, the photoreceptor-bipolar synapse. The synaptic disjunction has been conclusively documented in retinas days after detachment by electron microscopic examination of serial sections^12^. We have observed synaptic disjunction of rod-bipolar synapses after only 2 h of detachment using confocal microscopy^13^. Cone photoreceptors also respond to detachment and exhibit shape changes of their presynaptic pedicle and active zone, but they do not retract their terminals^14^. Besides the synaptic remodeling shown by the photoreceptors, other cells in the retina react as well. Bipolar and horizontal cells, for instance, sprout extensively into the outer nuclear layer (ONL)^15^. Reattachment of the retina does not fully repair these synapses. On the contrary, reattachment results in additional abnormalities, including sprouting of new neurites from rod photoreceptor terminals into the inner nuclear layer^16,17^. Histopathology of human retinas that have undergone retinal reattachment surgery shows very similar structural changes^18^. Thus, incomplete structural recovery has led us (and others^14^) to propose that synaptic changes contribute to the incomplete visual recovery observed in patients who undergo surgically successful retinal reattachment.

Our previous work with in vitro and in vivo RD models demonstrated a significant link between the activation of the RhoA pathway and rod axon retraction^13,19,20^. We showed that inhibition of activated Rho kinase (ROCK) by the ROCK inhibitors Y27632 and fasudil can reduce synaptic disjunction^13,21^. However, the effective concentration of these ROCK inhibitors was high, 1 and 10 mM, respectively, suggesting the possibility of off-target effects such as inhibition of protein kinase C or protein kinase A^22,23^. These concerns led us to evaluate AR13503, which is the active metabolite of Netarsudil^24^, a clinically-approved ROCK inhibitor. AR13503 inhibits both ROCK isoforms, ROCK 1 and 2, 100-fold more potently than Y23632 or fasudil and therefore could have high efficacy at lower doses^25^.

Here, we test whether AR13503 is effective in reducing rod-bipolar cell disjunction after RD and whether this inhibition improves retinal function 2 days after retinal reattachment. We tested ROCK inhibition in pigs because porcine eyes are similar to human eyes in size, retinal anatomy, and vasculature^26–28^. Pigs are diurnal, have both rod and cone photoreceptors, and the retina has an area centralis rich in cone cells that is similar in function to the macula in humans. Moreover, the porcine and human electroretinograms (ERGs) are similar^29,30^.

To investigate the potential for possible clinical translation, we chose: (1) to use small detachments, similar in size and height to iatrogenic detachments used for subretinal injection of stem cells and viral vectors^31–33^, and (2) to allow for spontaneous retinal reattachment, as often is done after such subretinal procedures. We show that the destructive structural and functional changes of the retina that occur after retinal injury are partially mitigated by the inhibition of ROCK activity. Thus, we propose a potential adjunctive therapy for iatrogenic detachments that uses ROCK inhibition to stabilize the synaptic circuitry of the neural retina. Stabilization of synaptic circuitry by ROCK inhibition may have wide application in other traumas and diseases of the central nervous system^34,35^.

## Results

### Subretinal administration of AR13503 decreased the amount of rod terminal retraction in 2-h detachments

AR13503, a ROCK 1 and 2 inhibitor, is the active metabolite of the FDA-approved netarsudil (AR13324) developed by Aerie Pharmaceuticals Inc. (Durham, NC). AR13503 has a Ki of 0.2 nM for both ROCK 1 and 2, and Ki’s of 1 nM and 27 nM for PKA and PKC, respectively^25^. It is likely to have higher efficacy in the eye than other ROCK inhibitors we have tested, as Y27632 has Ki’s of 22 nM and 41 nM, and fasudil has Ki’s of 76 nM and 47 nM for ROCK 1 and ROCK 2, respectively^25^.

**Figure 1:**
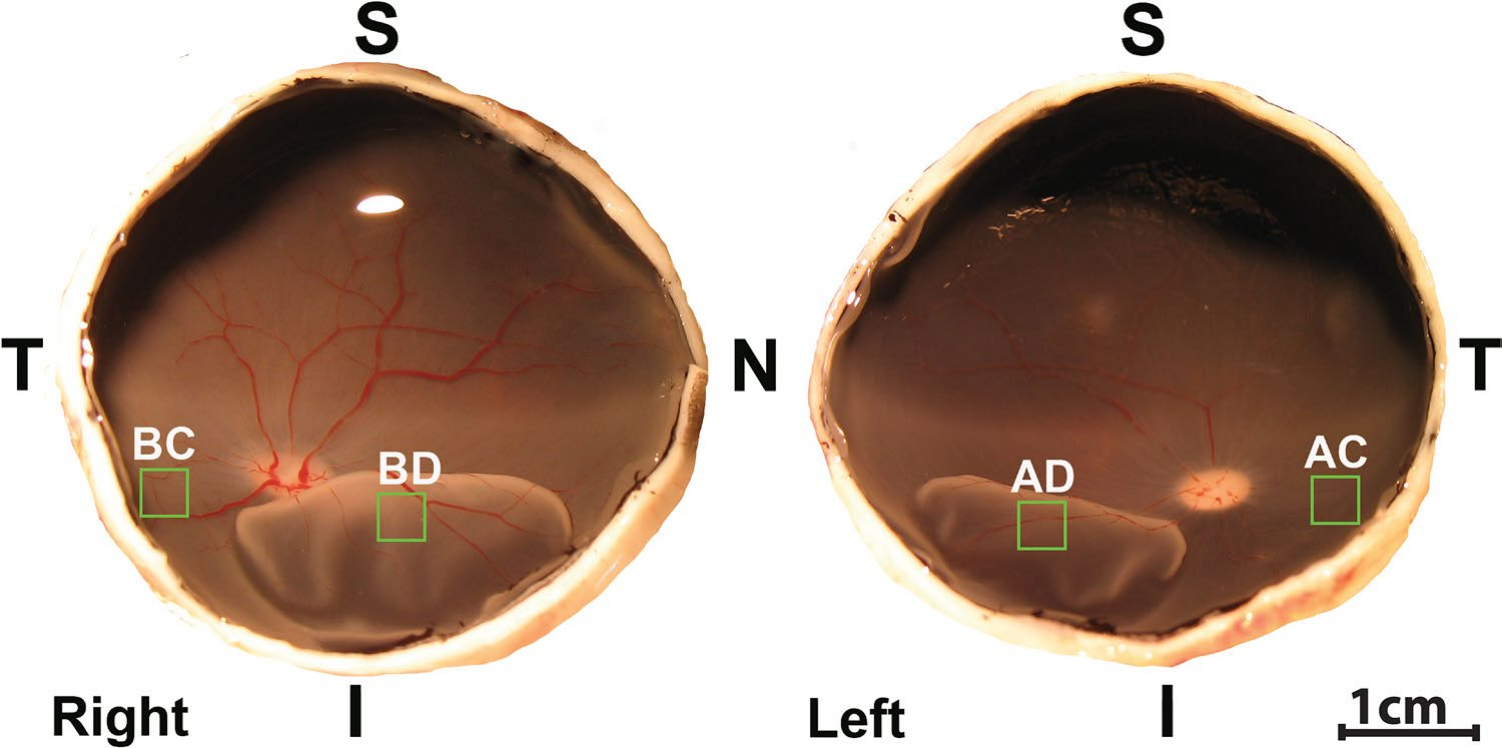
Eyecups from a right and left eye illustrating retinal detachments in the nasal-inferior quadrants. Location of retinal samples (green box) taken from BSS-treated eye attached (BC) and detached area (BD) and from the AR13503-treated eye corresponding attached (AC) and detached areas (AD). *S* superior, *T* temporal, *I* inferior, *N* nasal.

In order to compare the effects of AR13503 with our previous experiments using Y27632 and fasudil, we followed exactly the same protocols for the experimental RD and the morphological analysis of the retina as before^13,21^. We created detachments in both eyes of a single animal and treated 1 eye with subretinal injection of the ROCK inhibitor diluted in BSS and the fellow eye with BSS alone. We chose a concentration of 0.5 μM AR13503, in consultation with Aerie Pharmaceuticals. Once created, detachments remained for 2 h before euthanasia and enucleation. The presence of RD was confirmed after fixation and bisection of the eyes (Fig. 1). We examined retraction of rod spherules in confocal images of SV2-labeled retinal sections.

We have shown previously that SV2-immunolabeling in normal retina is observed only in the inner segments of photoreceptors and in the synaptic layers, i.e., the OPL and inner plexiform layers (IPL)^36^. However, after RD, SV2-labeling occurs in the ONL. Label in the ONL is due to retraction of the rod axon terminal and rearrangement of synaptic vesicles within the cells resulting in label in rod cell somata as well as in individual rod spherules^13,21^. Disconnection of the rod bipolar dendrites and the rod synaptic terminals in retina detached for 2 h has been described previously in our porcine retinal detachment model using rod bipolar and synaptic markers^13^.Thus, the synapses are broken. This interpretation is consistent with the phenomenon of synaptic disjunction observed in cat retinal detachment model using both light and electron microsocopy^11,12^.

**Figure 2:**
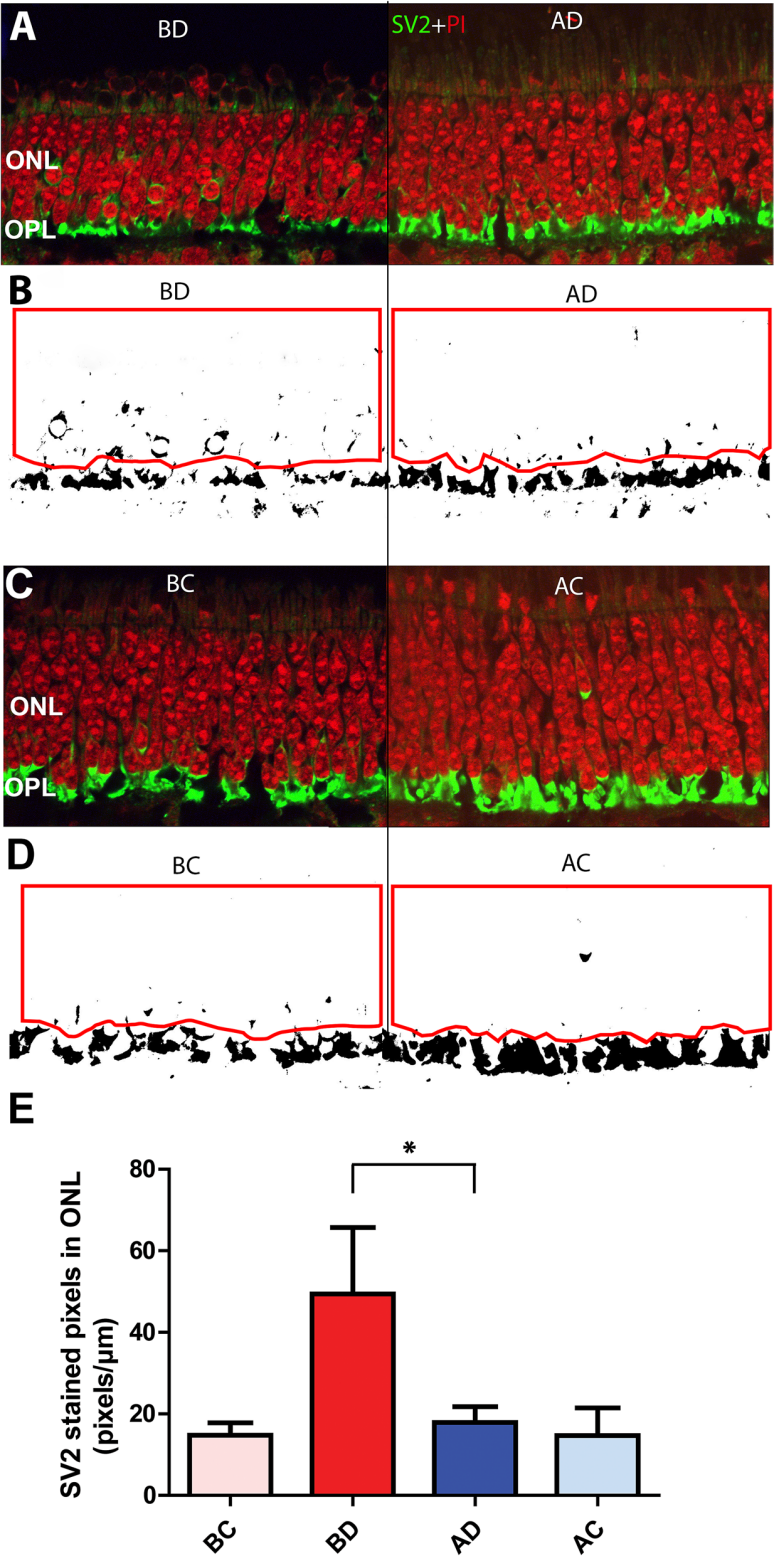
Effect of a subretinal dose of 0.5 μM AR13503 2 h after detachment. **(A,C)** Retina labeled for synaptic vesicle protein (SV2, green) and nuclei (propidium iodide, PI, red). Retracted rod spherules in the ONL are abundant in the sample from BSS eye detached area (BD)**. (B,D)** Binary mask created for SV2 channel for data analysis. Red line indicates the outline of the ONL, where the amount of SV2 labeling was measured. (**E)** There was significantly less rod spherule retraction in the AR13503-treated detached area (AD) compared to the detached area from the untreated eye (n = 3 animals, *p = 0.001, using 120 images/animal, + /− SD). BC, AC, attached areas of the untreated and treated retina.

We have described previously the spread of retraction to attached regions in the inferior part of the retina after 2-h detachments^13^. In line with this observation, in the present experiments, rod synaptic terminal retraction into the ONL, occurs in detached retina (BD, AD) and also in attached retina approximately 1 cm away, inferotemporally, from the detachment (BC, AC) albeit at lower levels (see Fig. 1 for location of sampling; Fig. 2A–D). Here, we also addressed the question of how far the injury spread superiorly after RD. Using eyes in which the detachments were made with BSS, samples were taken from the nasal- and temporal-superior (NS, TS) quadrants of the retina approximately  1 and 2 cms away from the edge of the RD. Rod synaptic terminal retraction was present in all sections; the level of retraction was less than in the detached area (BD) (BD = 55.4 ± 13.5; NS = 13.3 ± 3.4; TS = 14.4 ± 3.6; n = 4 animals, all in pixels/μm of ONL length, + /− SD). Normalizing the amount of retraction in the attached retina by looking at the amount of retraction in attached retina/amount in detached retina, it appeared that retraction in attached retina was about 25% of the amount in detached retina both in the inferior and superior attached retina. Thus, synaptic injury appears to occur extensively in the retina 2 h after detachment and at least as far away as 2 cms from the edge of the detachment.

AR13503 treatment significantly decreased the number of SV2-labeled pixels in the detached retina (AD) by 63.8% (n = 3 animals, p = 0.001) compared to the untreated detached retina (BD) (Fig. 2E). However, we found no difference between the drug-treated attached retina (AC) compared to the corresponding attached retina in the control eyes (BC). The reduction of retraction in the detached retina by 0.5 μM AR13503 (63.8%) was greater than either 1–10 mM Y27632 (34.5–43.7%^13^) or 10 mM fasudil (51.3%^21^).

Thus, we conclude that (1) the synaptic disjunction spreads in the retina well beyond the detachment, and it is not confined to the detached area; (2) the drug primarily reduced the synaptic damage in the bleb area where the injury was induced, and the drug was applied; (3) AR13503 is more efficacious than previously used ROCK inhibitors.

### Intravitreal administration of AR13503 decreased the amount of rod terminal retraction in 2-h detachments

Intravitreal injection of drugs is more straightforward clinically than subretinal injection because intravitreal injections can be done in an outpatient office setting whereas subretinal injections require surgery in an operating room. Therefore, we also administered AR13503 intravitreally at the time of retinal detachment to test for reduction of photoreceptor axonal retraction after RD. In order to compare with previous intravitreal injections of fasudil, we again followed our previous procedures ^21^. Final AR13503 concentrations in the vitreous cavity were calculated to be 0.5, 0.75, and 1.5 μM for these experiments.

**Figure 3:**
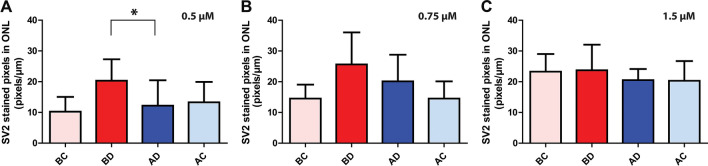
Effect of AR13503 intravitreal injection on axonal retraction of photoreceptors 2 h after retinal detachment. **(A)** 0.5 μM AR13503 treatment. There was a 40.2% decrease between the treated (AD) and the untreated (BD) detached areas (n = 3 animals, *p < 0.0001, using 120–180 images/animal, + /− SD). (**B)** 0.75 μM AR13503 treatment. Retraction was reduced by 21.6% in the treated detached area (AD) compared to the untreated detached area (BD) (n = 3 animals, p = 0.48, using 120–180 images/animal, + /− SD). (**C)** 1.5 μM AR13503 treatment. There was a 13.5% decrease in axonal retraction in the treated detached area (n = 4 animals, p = 0.43 using 120–180 images/animal, + /− SD). Only the 0.5 μM AR13503 dose showed a significant reduction in retraction. BC, AC, attached areas of the untreated and treated retina.

For 0.5 μM AR13503, there was a significant, 40.2% (p < 0.0001, n = 3 animals) difference between the extent of synaptic disjunction in the saline- and drug-treated detached areas (Fig. 3A). For the other doses, 0.75 and 1.5 µM AR13503, there was a reduction of 21.6% (n = 3 animals, p = 0.48) and 13.5% (n = 4 animals, p = 0.43) in SV2-labeled pixels in detached retinae; however, these reductions were not significant (Fig. 3B,C). The reduction in the detached retina by intravitreal 0.5 μM AR13503 was smaller than for subretinal injection of 0.5 μM AR13503.

These results indicated that immediate treatment with AR13503 via intravitreal injection reduces axon retraction after RD, primarily in the detached retina, and of the doses used, the 0.5 µM dose gave the best outcome.

### Histopathology in the outer retina after retinal detachment and spontaneous retinal reattachment

**Figure 4:**
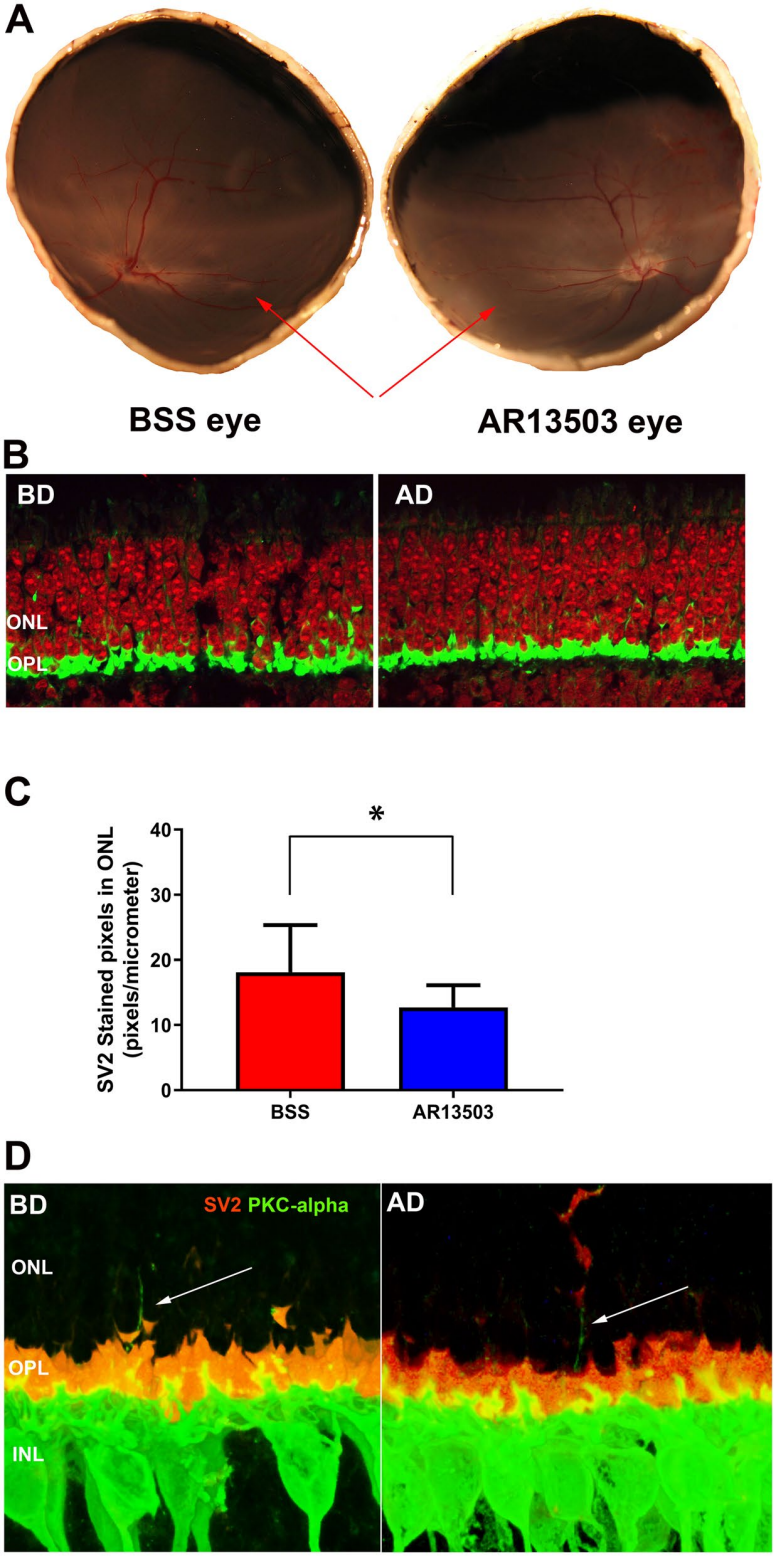
Morphological results 2 days after retinal detachment. **(A)** Detachments have spontaneously reattached (red arrows). (**B)** Retraction in the saline-treated eye detached area (BD) and the drug-treated eye, detached area (AD). Retina labeled for synaptic vesicle protein (SV2, green) and nuclei (propidium iodide, PI, red). (**C)** Axonal retraction was significantly reduced by 29.7% in AR13503-treated eyes compared to the eyes that received BSS alone (n = 6 animals, *p = 0.04, using 60 images/eye, + /- SD). Attached and detached areas of each eye were combined for this analysis (AC + AD vs. BC + BD). Data on the individual areas are reported in the Results. (**D)** Sprouting of bipolar cells. Retinae labeled for synaptic vesicle protein (SV2, red) and rod bipolar cells (anti-protein kinase C-alpha, PKC-alpha, green). White arrows indicate the fine dendritic processes of the bipolar cells extending into the outer nuclear layer (ONL). Pictures were taken of detached-spontaneously reattached areas both from BSS- (BD) and drug-treated (AD) eyes.

With 2-h detachments, the application of subretinal 0.5 µM AR13503 during or immediately after the creation of a RD was more efficacious than fasudil or Y27632 in reducing the disjunction of the rod-bipolar synapses in the detached retina by preventing rod terminal retraction. Thus, we pursued experiments at a longer time point using the same dose to test the efficacy of AR13503 over time. Two days after detachment, most detachments have reattached (Fig. 4A). Only animals with fully reattached retinae were used for analysis. This time point thus served not only to test for the longer-term effects of the drug, but it also allowed for iatrogenic detachments to reattach spontaneously.

In all eyes, treated and untreated, SV2 labeling in the ONL, indicating rod axon terminal retraction, is present at 2 days after reattachment (Fig. 4B). Drug-treated detached areas showed 36.6% less synaptic retraction in the ONL than BSS detached areas (BC = 13.8 ± 4.3; BD = 22.3 ± 12.6; AD = 14.1 ± 5.4; AC = 11.3 + /− 3.9; all in pixels/μm of ONL length, + /− SD; paired t-test, n = 6 animals, p = 0.047). In order to compare the morphology to the full-field ERG responses, which provides information from the entire retina, we also examined the average numbers of retraction in each eye. Samples from the detached and attached areas of drug-treated eyes showed significantly less SV2 labeling in the ONL by 29.7% vs. the combined corresponding areas from the BSS eyes (Fig. 4C. combined BSS = 18.1 ± 7.2; combined AR13503 = 12.7 ± 3.4; all in pixels/μm of ONL length, + /-SD; n = 6 animals, p = 0.04).

Although SV2 labeling in the ONL is still present at day-2, we noted that the amount of retraction was less than in the sections from 2-h RDs. Statistical analysis showed that the BSS detached-reattached area had a significant 54.9% decrease in pixels in the ONL at day-2 compared to the 2-h detachment (BD = − 26.65 (-54.9%); average reduction in pixels/μm, n = 9 animals, p = 0.003, using 144 sections, 1080 images). Other areas showed slight decreases in the number of pixels, indicating rod photoreceptor synaptic terminal retraction in ONL, but these decreases were not significant (BC = − 0.76 (− 6.0%), p = 0.71; AD = − 4.19 (− 21.0%), p = 0.11; AC = − 3.67 (− 23.3%), p = 0.33; average reduction in pixels/μm of ONL length, reduction in percentage).

One plausible explanation for a decrease in SV2-labeled pixels in the ONL would be the death of rod photoreceptors. One of our earlier observations was that when the pig retina is detached for 4 h, rod photoreceptor cell death is present^21^. However, in the current 2 h and 2-day retinal samples we did not encounter pyknotic rod photoreceptor nuclei suggesting that reduced labeling is not due to cell death. Another plausible explanation for a decrease in SV2-labeled pixels can be a reduction in synaptic proteins or vesicles over time. The amount of total labeling in the outer retina (OPL plus ONL) was measured and compared in the 2-day and 2-h samples (n = 9 animals). There was no statistically significant difference in the number of labeled pixels in the outer retina over time, thus a decrease in synaptic proteins as a possible reason for a reduction of pixels in the ONL after 2 days is unlikely (data not shown). Rather, this result suggests there is movement of some SV2 protein/synaptic vesicles from the ONL back to the OPL.

In addition to rod photoreceptors, other cell types in the retina also react to the detachment injury. In particular, Lewis et al. ^15^ demonstrated that rod bipolar dendrites sprout into the ONL after detachment. Whether this occurred simultaneously with rod axon retraction was not known. Previously, we found that the rod and bipolar cell connection is disrupted when the retina is detached for 2 h^13^. At that timeframe, there was no evidence of a bipolar reaction. In our current 2-day experiments, we did observe occasional thin, hair-like sprouts from rod bipolar cells, identified by their PKC-alpha labeling (Fig. 4D). In some cases, the sprouts contacted SV2-labeled terminals. This sprouting was present in all eyes, both the BSS- and AR13503-treated eyes, and in both the detached and attached areas. The occurrence of sprouts was too infrequent (11 sprouts in 10.2 cm of examined retina, n = 1 animal) to be able to quantify any differences across the areas.

Thus, retraction is reduced but remains an important finding 2 days after detachment/reattachment, and treated eyes still had less synaptic disruption. In addition, rod bipolar dendritic sprouting is present by 2 days after detachment even in the presence of retinal reattachment.

### AR13503 improved the functional outcome after retinal detachment and spontaneous reattachment

**Figure 5:**
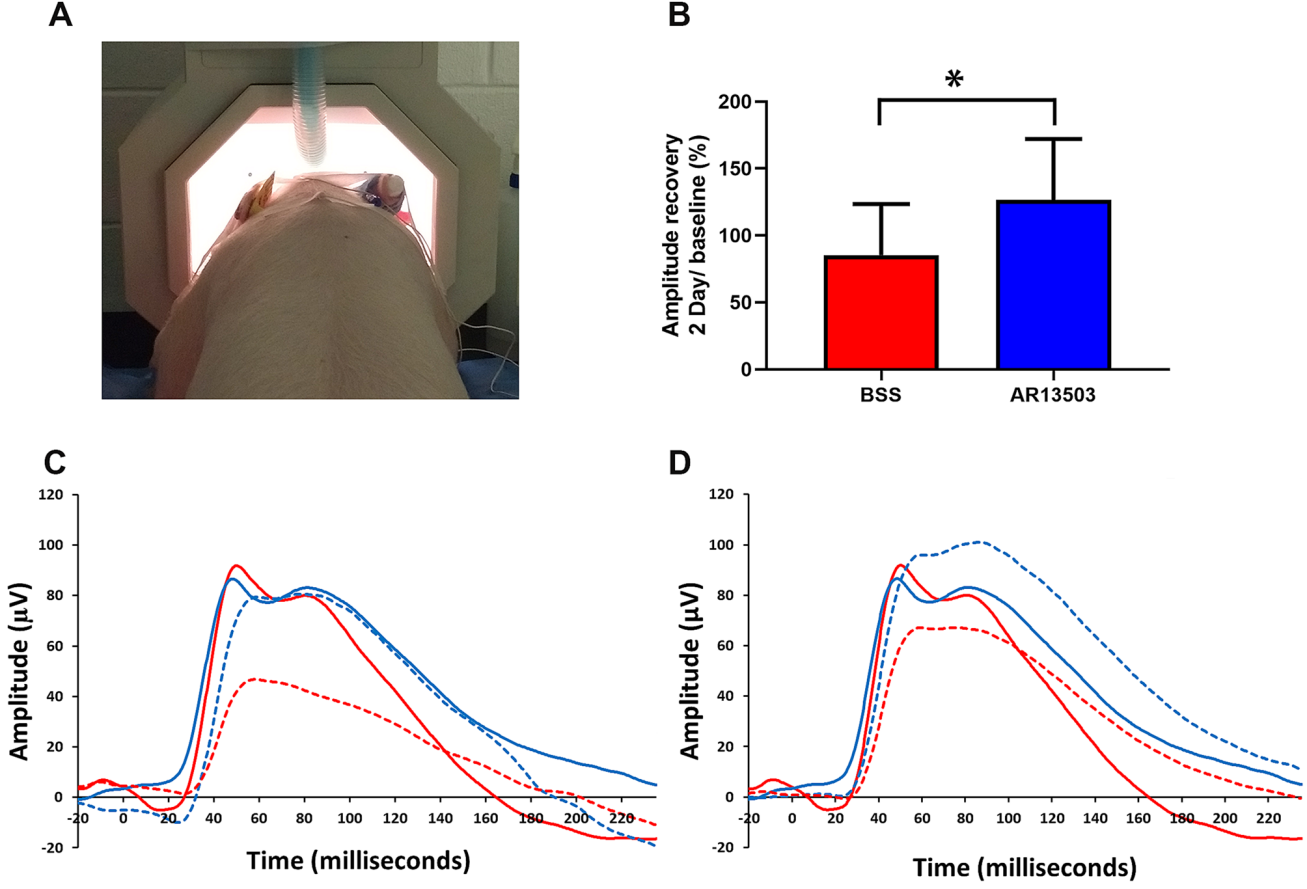
Electrophysiological results 2 days after retinal detachment of eyes with spontaneous retinal reattachment. **(A)** Position of pig in the Ganzfeld stimulator. (**B)** There was a 48.6% difference in amplitude recovery between the AR13503-treated eyes, subretinal injection of 0.5 μM, compared to the eyes that received BSS alone (paired t-test, n = 5 animals, *p = 0.017, + /- SD). (**C,D)** Representative waveforms of rod-specific scotopic responses (0.01 cd s m^-2^). Red lines, untreated eye; blue lines, AR1303-treated eye. Solid line, preoperative response; dashed line, postoperative response. (**C)** At 2 days in the treated eye (blue solid and dashed lines) the evoked responses recovered to the baseline levels. However, in the untreated eye (red solid and dashed line) the response was lower than the recorded preoperative responses. (**D)** Representative waveform showing supernormal (higher than baseline) response recorded from treated eye (blue dashed line) at 2 days.

Because AR13503 continued to show a significant reduction of synaptic retraction 2 days after injection, we tested for possible functional differences in treated versus untreated retinae. Full-field dark-adapted ERG responses were recorded preoperatively (as baseline) and 2 days later. We focused on scotopic responses to test for rod cell function specifically. To account for variability between animals and between eyes within an animal, amplitudes were normalized as percent of baseline for each eye. Functional outcomes were in line with the morphological results as the b wave amplitude, an indication of the level of transmission between rod photoreceptors and ON-bipolar cells, was improved by 49% at 2 days in the AR13503 treated eyes compared to the BSS eyes (Fig. 5B paired t-test, n = 5 animals, p = 0.017). In 3 of 5 animals, the amplitude recovery not only reached the baseline level but exceeded the preoperative baseline after reattachment. Representative waveforms are shown in Fig. 5C,D. ERGs with larger than normal amplitudes have been termed, “supernormal ERGs”^37^. This phenomenon occurred mainly in the AR13503 treated eyes (3 of 4 eyes showing supernormal ERGs were treated with ROCK inhibitor). The implicit times were similarly delayed in the BSS- and the AR13503-treated eyes by 2 days compared to the preoperative (baseline) status, with an average of 8.7 ms (BSS, n = 5 animals, p = 0.005) and 9.0 ms (AR13503, n = 5 animals, p < 0.0001) respectively. Such delays would suggest that synaptic transmission between photoreceptors and rod-driven bipolar cells was not fully restored. However, based on response amplitude, the data suggest that ROCK inhibition treatment improves the functional outcome measured by ERG at 2 days.

### Relationship between structure and function at 2 days

**Figure 6:**
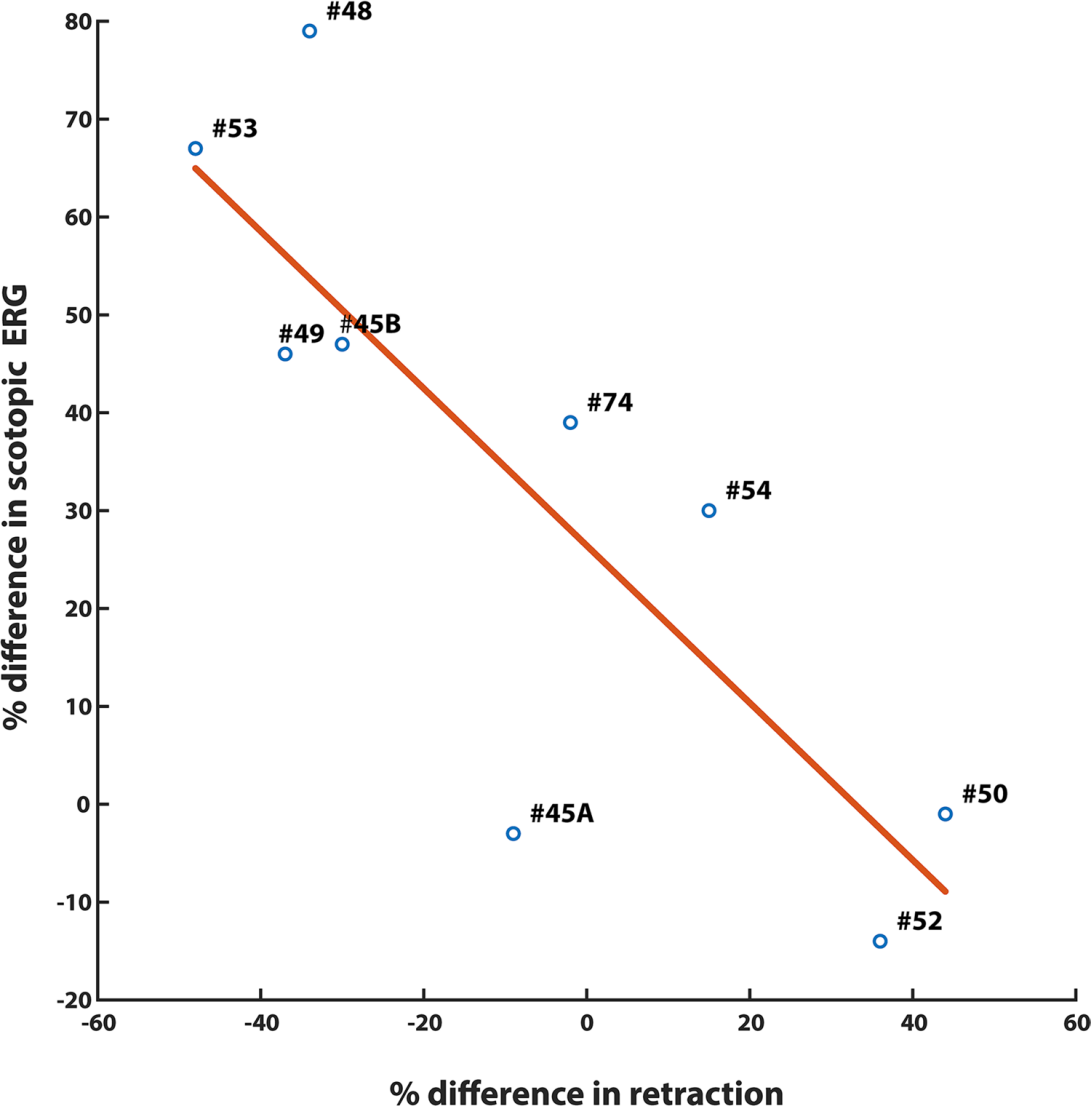
Correlation between the morphological and functional outcomes 2 days after injury. Linear regression model was applied to n = 9 animals. The dose of the drug (0.5 μM, n = 6 animals, and 25 μM, n = 3 animals) and the size of the retinal bleb (quarter-, n = 8 animals, or half of the retina, n = 1 animal) varied among these animals; a correlation between the anatomical and the functional change is present. (#numbers are identification numbers for individual animals, see Suppl. Table 1).

In general, reduced retraction in the detached retina appeared to correlate with increased scotopic ERG responses. To examine this relationship more broadly, we used all eyes that had reattached retinas and in which we recorded ERG scotopic responses after 2 days and analyzed retinal sections. Thus, we included animals that were not included in the analyses of drug efficacy because of the high dose used (animals #52, #53, #54) or the large size of the detachment (animal #52) or because the data were considered to be outliers (animal #50) (see Supplementary Table 1). We calculated the inter-eye differences within each animal for both morphology and ERG using averaged data (#-animal ID in Suppl. Table 1; percent difference in pixels/um and percent difference in ERG recovery; #45A = − 9/− 3; #45B = − 30/ + 47; #48 = − 34/ + 79; #49 = − 37/ + 46; #50 =  + 44/− 1; #52 =  + 36/− 14; #53 = − 48/ + 67; #54 =  + 15/ + 30; #74 = − 2/ + 39; Fig. 6).

For morphological data, negative values indicate a reduction, and positive values indicate an increase in synaptic damage in the drug-treated eye compared to the control eye. For the ERG, negative values indicate deterioration, and positive values indicate an improvement in amplitude of the scotopic rod-specific response in the drug-treated eye compared to the BSS eye. Thus, in animals where a minus for morphological data is coupled with a plus for ERG values, the reduction in synaptic retraction was accompanied by improvement in rod-driven function. There was a negative correlation between the 2 variables (r^2^ = 0.681, n = 9 animals, p = 0.006), suggesting that a decrease in synaptic damage by ROCK inhibition improves retinal function. When we use the morphological values from the detached area only (#-animal ID in Suppl. Table 1; percent difference in pixels/μm and percent difference in ERG recovery; #45A =  + 20/− 3; #45B = − 53/ + 47; #48 = − 33/ + 79; #49 = − 13/ + 46; #50 =  + 10/− 1; #52 =  + 18/− 14; #53 = − 18/ + 67; #54 = − 17/ + 30; #74 = − 30/ + 39), the correlation is similarly strong (r^2^ = 0.652, n = 9 animals, p = 0.009). Although this correlation does not capture within eye and within animal variability, overall the relationship suggests that synaptic damage in the OPL is correlated with scotopic function. Moreover, it suggests that reducing retraction by ROCK inhibition may improve the outcomes of iatrogenic detachment/reattachment.

## Discussion

Our previous in vivo work showed that RhoA activation occurs in the pig retina within 2 h after retinal injury and that ROCK inhibition can mitigate injury-related synaptic disjunction in the OPL^13,21^. However, both previously used ROCK inhibitors required high (millimolar) concentrations. Here, AR13503 was effective at a more than 1000 times lower dose than fasudil or Y27632. High millimolar doses could mean there are non-specific effects associated with the synaptic rescue, however, the efficacy of the nanomolar dose of AR13503 indicates that specific effects against ROCK are the primary cause of the inhibition of synaptic disjunction in the detached retina.

Although both subretinal and intravitreal injection of 0.5 µM AR13503 were effective in the detached retina, subretinal injection showed greater inhibition of axonal retraction. This result may be due to different patterns of diffusion. During subretinal injection, the AR13503 solution is delivered directly to the site of the injury in a relatively limited space between the photoreceptors and the RPE, whereas after intravitreal injection, the drug can disperse widely and has more cellular layers to diffuse through to reach the photoreceptors.

We have reported that RhoA activation increases significantly in the adjacent attached retina^13^, and our previous reports^13,21^ as well as current results show widespread synaptic disjunction in attached regions of the retina in 2 h, even with these relatively small detachments. The reason for the widespread synaptic damage after injury is not known. Others have also reported changes in the attached retina including upregulation of GFAP^38^, upregulation of inflammatory and immune-response related genes^39^, and proliferation of retinal cells^40^ although the timing of the changes and the methods of retinal injury varied from ours. Mechanical- or ischemia-induced spreading depression is well-known after brain trauma^41–43^. A similar phenomenon occurs in the retina^44^. We suggest that spreading depression may cause the wide-spread activation of RhoA and resulting rod axon retraction in the retina.

For the attached retina, there was no significant effect of ROCK inhibition on rod axon retraction. It is possible that diffusion again plays a role in these results. Transport properties of the RPE in detached and attached retina differ, which could cause more fluid flow across the detached versus the attached retina^45^. Previously we demonstrated that high doses (10 mM) of Y27632 showed significant reduction of retraction in attached retina^13^. High doses may increase the chance of obtaining effective drug levels in the attached retina. Alternatively, the effect could be due to some additional non-specific effect on another kinase, perhaps PKC. In other words, we cannot rule out the possibility that the pathways for retraction might be slightly different for detached and attached retina.

After 2 days, detached retina reattached spontaneously. However, retracted rod terminals remained in both previously detached and attached retina. It is known that some pathologies caused by detachment do repair over time following retinal reattachment. For example, the photosensitive outer segments are restored after reattachment^46^. But, consistent with our observations, disjunction of the rod photoreceptor-bipolar synapse is still apparent after the detached retina has been reattached. Fisher et al.^16^ documented synaptic retraction in cat retina 28 days after reattachment of a 3-day detachment. In humans with RD not involving the fovea, approximately 25% have vision 20/40 or worse^7^. We speculate that among other causes, such as cystoid macular edema, macular hole, and epiretinal membrane formation, synaptic disjunction may contribute to suboptimal visual outcome, and its occurrence in fovea-sparing detachments may be due to spreading depolarization.

Fisher and colleagues^15^ also described sprouting from rod bipolar cells into the ONL after detachment. Rod bipolar sprouting, as well as rod terminal retraction, have now also been observed in mouse models of retinal degeneration^47,48^. It has been suggested that as rod terminals retract, they “pull” the rod bipolar dendrites with them into the ONL^47^. However, Linberg et al.^12^ identified many rod terminals unconnected to rod bipolar dendrites after a 1-week detachment. Previously, we reported that rod axon retraction was observed 2-h after detachment in the absence of bipolar sprouting ^13^, indicating that the rod-to-bipolar junction had separated. In the present experiments, 2 days after detachment followed by retinal reattachment, a few bipolar sprouts in contact with retracted terminals were observed, but most terminals were unattached to bipolar cells. It seems that after detachment, synaptic disjunction is the more prevalent initial event and that bipolar dendritic sprouting and contact with retracted presynaptic terminals is a subsequent event.

We did observe some significant reduction in synaptic protein labeling in the ONL after spontaneous reattachment of detached retina in untreated eyes, which suggests that some axonal retraction was reversed. This return of synaptic protein/vesicles to the OPL may be part of the growth of rod neuritic processes after reattachment observed by Fisher et al.^14,16,17^. Indeed, a month after reattachment of a 3-day detachment, neuritic processes from rod cells grow into the INL, in a manner consistent with other retinal diseases that affect rod cells^49,50^. To the best of our knowledge, the degree to which rod-bipolar synapses recover after retinal reattachment remains to be investigated. In ROCK-inhibited eyes, no significant change in levels of retraction was observed suggesting that the synaptic circuitry was more or less stable.

Drug treatment reduced retraction, compared to control, over the 2-day period we examined. Anatomical savings in the rod-bipolar synapse were coupled with improved rod-specific scotopic amplitudes in the treated eyes measured by ffERG. One surprising finding was the larger than baseline b-wave amplitudes at 2 days, termed "supernormal ERGs” in the earliest observations of large scotopic b-waves^37,51^, primarily in the ROCK-inhibitor-treated eyes. Although increase in ERG amplitudes is uncommon among visual disorders, such observations have been reported in patients with cone dystrophies^37,51,52^, early diabetic retinopathy^53^, central retinal vein occlusion (CRVO)^54,55^ and in animal disease models, e.g., Ant1-deficient mice^56^, a rabbit model of retinal degeneration^57^, and a rat traumatic brain injury model^58^. In normal porcine retina, intravitreal injection of AR13503 alone did not cause increased scotopic ERG responses after 7 days (n = 3 animals, unpublished data).

Although the mechanism for supernormal ERGs is unknown it has been suggested to be the result of the following. (1) An imbalance of the retinal excitatory and inhibitory signaling, in particular the lack or diminished presence of inhibitory signaling^58–60^. (2) Increased nitric oxide (NO)^61^. ROCK inhibition increases the phosphorylation of endothelial nitric oxide synthetase (eNOS), increasing NO production^62,63^. Thus, increased NO may underlie the development of supernormal ERGs in the ROCK-inhibited eyes. Or (3) VEGF levels in the retina. VEGF is known to increase eNOS and NO levels in the retina^64^. The VEGF effect may occur via VEGF receptor 2 activation and production of the classical NO effector cGMP^65,66^. Kroll et al.^67^ suggested that ROCK inhibition enhanced the activation of VEGF receptor 2. Thus, VEGF may contribute to supernormal responses in the ROCK-inhibited eyes via VEGF receptor-induced increases of NO. However, it should be noted that the link between VEGF and ROCK inhibition continues to be investigated.

Whether supernormal ERGs in our ROCK-inhibited eyes mean better vision in the long-term is unknown, as the survival time in our experiments was quite short. However, work in patients is encouraging. Miyata et al.^55^ suggested that after anti-VEGF treatment of non-ischemic CRVO, eyes with supernormal ERGs had a better prognosis after 1 year than non-ischemic CRVO eyes without supernormal ERG amplitudes.

This study has limitations. First, for the detachment-spontaneous reattachment experiments we do not know exactly when the retinae reattached. One of our earlier findings was that when the pig retina is detached for 4 h, rod photoreceptor cell death is abundant^21^. Since we did not encounter pyknotic photoreceptor nuclei in the present study, we speculate that the retina reattached within the first 4 h after the blebs were created. Second, at this time we have focused only on the anatomical and electrophysiological changes occurring in the rod-bipolar synapse. In the future, we will examine how other cell types behave in this iatrogenic RD model and whether ROCK inhibition affects them.

Therapeutic approaches for subretinal delivery of stem cells, viral vectors, or visual prostheses involve iatrogenic RDs. In some cases, iatrogenic detachment involves active surgical reattachment^32,68,69^; in other cases, the retina reattaches spontaneously^33,70,71^. Our evidence indicates that even brief, relatively small detachments cause synaptic damage that spreads throughout the retina and reduces retinal function, at least at the 2-day time point. There are undoubtedly multiple reasons for poor visual recovery in therapies involving iatrogenic detachment^31–33^. We suggest that the addition of ROCK inhibition subretinally or intravitreally during or perhaps prior to the detachment may improve post-procedure outcomes.

In addition to RD, there is a list of retinal disorders that exhibit rod synaptic terminal retraction: age-related macular degeneration^72,73^; rat models of glaucoma^74^, retinal degeneration^75^, and oxygen-induced retinopathy^76^; mouse models of retinoschisis^77^ and congenital stationary night blindness^47^. If the synaptic pathology is caused by RhoA activation in these disorders, ROCK inhibition could be beneficial for a broad spectrum of retinal disease.

Our findings may also be relevant more generally to CNS injury. In traumatic brain injury (TBI) for instance, there can be extensive damage to synaptic connections^35,78–80^ resulting in synaptic loss as well as structural remodeling of dendritic spines^35,78^. The synaptic damage extends beyond the immediate area of trauma^81^. The downstream effectors of RhoA contribute to synaptic plasticity^82^, and RhoA is also upregulated in brain injury^83,84^. Thus, it seems reasonable to suggest that ROCK inhibition can prevent synaptic disjunction in the brain after trauma. Indeed, the use of the inhibitor fasudil has already been successful in preventing synaptic damage and restoring function in a rodent model of TBI^35^. Further, our model of RD is poised to be a useful scenario in which to test RhoA-ROCK inhibition in CNS injury. We have tested, for example, the inhibition of a RhoA downstream effector LIMK that showed effects as robust as ROCK inhibition in RD^85^. Continued investigations to rescue synaptic circuitry after retinal injury may contribute to potential therapies for TBI-related and other neurodegenerations.

## Materials and methods

### Animals

Three-month-old female Yorkshire pigs, weighing 30 kg, were obtained from Animal Biotech Industries (Danboro, PA, USA) and kept on a 12-h light/12-h dark cycle for at least 1 week prior to use. Animals were housed in an Association for Accreditation and Assessment of Laboratory Animal Care (AAALAC)-accredited pathogen-free facility, 1 animal to a pen. They were subject to overnight fasting with access to water ad libitum before surgery. Experimental procedures and methods of euthanasia were approved by the New Jersey Medical School Institutional Animal Care and Use Committee and adhered to the ARVO Statement for the Use of Animals in Ophthalmic and Vision Research. A total of 22 animals and 44 eyes were used. Further description of the animals can be found in Supplementary Table 1.

### Retinal detachment and experimental design

RDs were created under general anesthesia. Animals were injected with atropine (0.02 mg/kg; VetUS, Henry Schein, Dublin, OH, USA) and sedated with an injection of ketamine (20 mg/kg; Mylan Institutional LLC, Galway, Ireland) and xylazine (2.2 mg/kg; Lloyd Lab., Shenandoah, IA, USA), all administered intramuscularly. After 5–10 min, a peripheral venous catheter was inserted through the auricular vein, and the animal was intubated with an endotracheal tube. To maintain anesthesia, the animals were supplied with 0.5% to 3.0% isoflurane in oxygen using a ventilator. Lactated Ringer’s solution was infused intravenously at a rate of 8 mL/kg/h. Vital signs (oxygen saturation, heart rate, and body temperature) were monitored and maintained within the normal range throughout the experiment.

For surgery, pupils were dilated with topical application of 1% Tropicamide (Bausch&Lomb, Tampa, FL, USA) and 2.5% phenylephrine (Paragon Bioteck, Portland, OR, USA). A standard 3-port vitrectomy was performed using 20-g instrumentation. The posterior hyaloid was detached over the area centralis using active suction and a core vitrectomy was completed. During and after vitrectomy, the vitreous cavity of the eye was perfused with balanced salt solution (BSS; Alcon, Fort Worth, TX, USA) containing 2 µg/mL epinephrine (Henry Schein, Dublin, OH, USA). A 33-g metal cannula was used to slowly inject BSS or Rho kinase inhibitor, AR13503 (Aerie Pharmaceuticals, Durham, NC, USA) dissolved in BSS, subretinally to create a RD (~ 10–15 mm in diameter) in the inferior nasal quadrant (Fig. 1). For intravitreal administration of drug, 150 µL of 10, 15, or 30 µM AR13503 dissolved in BSS was injected with a 30-gauge needle into the vitreous cavity (entering ~ 3 mm posterior to the limbus). Immediately after the procedure, the sclerotomies were closed with 7–0 vicryl suture. The volume of the vitreous cavity was calculated to be ~ 3 ml, and the final intravitreal concentrations were estimated to be 0.5, 0.75, and 1.5 µM, respectively.

After RDs were created the animals survived for an additional 2 h or 2 days. For the 2-h procedures, animals were kept under anesthesia for the 2 h after detachments were made and then euthanized with 7 ml intravenous Euthasol (Vibrac AH, Fort Worth, TX, USA) for enucleation. For the longer survivals, the conjunctiva was sutured after the sclerotomies were closed, 1.6 mg (0.4 ml) Dexamethasone (Fresenius Kabi, Lake Zurich, IL, USA) and 0.1 g (0.5 ml) Cefazolin (WG Critical Care, LLC, Paramus, NJ, USA) were injected subconjunctivally, and Tobradex ointment (Alcon, Fort Worth, TX, USA) was applied topically. Once the animals had recovered, they were maintained in their cage, with constant monitoring, for an additional two days. Animals were administered pre- and postoperative intramuscular injections of buprenorphine (0.01–0.05 mg/kg; Reckitt Benckiser HealthCare, Hull, England) and enrofloxacin (10 mg/ kg; Bayer HealthCare, Shawnee, KS, USA). At the 2-day time point the animals were again anesthetized, using the previous protocol, for ERG recording and structural analysis by fundus photography and optical coherence tomography (OCT) before being euthanized with 7 ml intravenous Euthasol for enucleation.

### Full-field flash electroretinogram (ffERG), fundus photography, optical coherence tomography (OCT)

The procedures for recording ffERGs, fundus photography, and OCT were done under general anesthesia, as described above. For all 3 procedures pupils were dilated and accommodation relaxed with topical applications of 1% Tropicamide and 2.5% phenylephrine hydrochloride drops. Adjustable lid specula were used to keep the eyelids separated. ERGs were recorded in animals that had 2-day survivals both before retinal surgery and 2 days after surgery. Fundus photography and OCT were performed in the animals 2 days after retinal surgery to confirm the status of the retina.

During electroretinography, flashes were produced and responses recorded using a UTAS ERG system with a BigShot Ganzfeld stimulator (LKC Technologies, Inc., Gaithersburg, MD, USA). The pig’s head was placed inside of the ganzfeld bowl (Fig. 5A), and bilateral ERGs were recorded simultaneously using ERG-Jet electrodes (Fabrinal SA, La Chaux-de-Fonds, Switzerland) placed on the cornea. The cornea was kept moist with a hypromellose ophthalmic demulcent solution 2.5% (Akorn Inc, Lake Forest, IL, USA). The reference electrode was placed at the midline of the forehead, about the same distance from both eyes. The ground electrode was placed in midline on the back between the shoulders of the animal. The stimulus protocol was based on the International Society for Clinical Electrophysiology of Vision (ISCEV) standard for clinical ffERG^86^. Briefly, after 30 min of dark adaptation, the ffERG was recorded to strobe flash intensities of 0.01 cd s m^−2^ with an interstimulus interval (ISI) of 2 s (15 samples) to isolate the rod scotopic response. A notch filter (60 Hz) and 85 Hz low pass filter were applied during data analysis using Matlab (The Mathworks, Natick, MA, USA) to eliminate noise and the oscillatory potentials. The amplitude and implicit time were measured from stimulus onset to b-wave peak, datapoints were automatically identified and values were calculated by custom made script in Matlab (The Mathworks, Natick, MA, USA). Individual responses were analyzed, and aberrant waveforms rejected before averaging.

### Sample preparation and immunohistochemistry

After enucleation the eyes were immersed in 4% paraformaldehyde (EMS, Hatfield, PA, USA) for 15 min; a 5 mm slit was made at the ora serrata to aid in rapid fixation. The eyes were then opened; the anterior segment and any remaining vitreous humor were removed carefully, and eyecups fixed overnight at 4 °C. Samples were collected from areas of retina that had been detached and from areas of the retina that had not been detached as diagrammed in Fig. 1. Retinae were immersed in 30% sucrose overnight at 4 °C. On the consecutive day, specimens were embedded in OCT compound (Sakura Finetek, Torrance, CA, USA) at room temperature for 2 h, then frozen and cut into 25-µm-thick sections using a cryostat, as described previously^13^.

Procedures for immunolabeling were as previously described^87^. Briefly, sections were washed 2 times with 0.3% Triton X-100 in PBS, blocked with 10% blocking buffer for 1 h at room temperature, and then incubated either in antibody for SV2 (1:100 dilution, Developmental Studies Hybridoma Bank, Iowa City, IA, USA) or antibody for PKC-alpha (1:100 dilution, Cell Signaling Technology, Boston, MA, USA) overnight at 4 °C. The next day, the sections were washed 3 times with 0.3% Triton-100 in PBS and incubated with secondary antibodies conjugated to Alexa Fluor 488, 546, or 647 (1:100 dilution, Life Technologies, Norwalk, CT, USA) for 90 min at room temperature, followed by nuclear staining with 1 µg/mL propidium iodide (1:100 dilution, PI; Sigma- Aldrich, St. Louis, MO, USA) or TO-PRO3 (1:500 dilution; Life Technologies, Norwalk, CT, USA) for 5 min at room temperature. After 2 washes with 0.3% Triton-100 in PBS, sections were covered with Fluoromount-G medium (SouthernBiotech, Birmingham, AL, USA) and preserved under coverslips sealed with nail polish. For all immunohistochemistry, sections from retinal areas to be compared were placed on a single slide so that they were labeled together, under the same conditions; control sections were also processed simultaneously with experimental sections but without primary antibodies.

### Quantification of axonal retraction

All data were collected by persons masked to the sample identifications. Sections were examined using confocal microscopy (model LSM510; Carl Zeiss Microscopy, Jena, Germany) by scanning 1 µm optically thick sections with a 63 × oil immersion objective. Brightness and contrast were set to obtain unsaturated images. Laser power and scanning rate were unchanged throughout a single experiment. Enhancements in brightness and contrast were performed (Photoshop 7.0 software; Adobe, CA, USA) only for presentation purposes^13^.

Two samples (BC, BD or AC, AD) from each eye, four samples per animal, were obtained (Fig. 1); 30–45 images were taken of each retina sample, and data were collected from two to four sections per sample, examining at least three different areas of each section. SV2 immunolabeling in the ONL was analyzed as described^13^. Briefly, a binary mask of the green channel was created for each image, the ONL was outlined using the PI labeled image as a guide, and the pixels in the ONL of the binary image were counted using ImageJ software (v1.45s; NIH). The measurements are reported as pixels per micrometer of ONL length.

To quantify total SV2 labeling in the outer retina a similar method was used: after the binary mask was created, both the ONL and the OPL were outlined, and pixels in the outlined area were counted.

### Statistical analysis

For statistical analysis Student’s t-test and generalized estimating equation (GEE^88^) were used. Normality was tested using the Shapiro-Wilks test. Use of the paired t-test was based on the experimental design, one eye was treated, and the other was untreated. Eyes were randomized for BSS or AR13503 treatment. Generalized estimating equation (GEE) was applied to estimate the parameters of a linear model with a possible unknown correlation between outcomes. To capture the strength of the relationship between the anatomical and the functional outcomes, we estimated a Pearson’s correlation coefficient from the average change in scotopic ERG and the average change in retraction. While the number of experimental units (animals) was at most 3 to 6 in each experiment, the number of outcomes for each eye-treatment-time combination was large and thus the use of large sample methods that adjust for intraclass correlation, e.g., GEE, is justified.

Statistical analysis was performed with GraphPad Prism 5.1, Matlab (The Mathworks, Natick, MA, USA) and SAS (Version 9.4)*.* The graphics were produced using GraphPad Prism 5.1 and Matlab (The Mathworks, Natick, MA, USA). Data are expressed as mean + /− the standard deviation (SD). We set alpha (type I error rate) at 0.05. Reported p-values were obtained via GEE analysis unless otherwise noted.

## Supplementary Information

Supplementary Information

## Data Availability

All relevant data generated or analyzed during this study are included in this manuscript and the supplementary information. Raw data can be obtained from corresponding author.
