# Supplementary material for: ROCK inhibition reduces morphological and functional damage to rod synapses after retinal injury
Source: Sci Rep. 2021 Jan 12;11:692. doi: 10.1038/s41598-020-80267-4 (PMC7804129; doi:10.1038/s41598-020-80267-4)
Supplement: Supplementary file 1 — Supplementary Information [file 41598_2020_80267_MOESM1_ESM.pdf]

Title: ROCK inhibition reduces morphological and functional damage to rod synapses after retinal injury

Authors

1. Éva Halász[1]

2. Marco A. Zarbin[2]

3. Amy L. Davidow[3]

4. Laura J. Frishman[4]

5. Peter Gombkoto[5]

6. Ellen Townes-Shouldbe [1,2]

[1] Department of Pharmacology, Physiology and Neuroscience, Rutgers New Jersey Medical School, 185 South Orange Avenue, Newark, New Jersey, 07103, United States.
[2] Institute of Ophthalmology and Visual Science, Rutgers New Jersey Medical School 90 Bergen Street, Newark, New Jersey, 07103, United States.
[3] Department of Biostatistics and Epidemiology, Rutgers School of Public Health, 683 Hoes Lane West, Piscataway, New Jersey, 08854, United States.
[4] College of Optometry, University of Houston, 3507 Cullen Blvd, Houston, Texas, 77204, United States.
[5] Center for Molecular and Behavioral Neuroscience, Rutgers University, 197 University Avenue, Newark, New Jersey, 07102, United States.

Correspondence and requests for materials should be addressed to E.T.-A. (email: andersel@njms.rutgers.edu)

Animal ID	Notes	Used/not used
Subretinal 0.5 μM (2 hrs)		
#41	depigmentation in od	
#42A		
#42B	depigmentation in ou	
#43A	RD reattached by 2 hrs in os (BSS)	Data not used
Intravitreal 0.5 μM (2 hrs)		
#43B	depigmentation in ou	
#44A		
#44B		
Intravitreal 0.75 μM (2 hrs)		
#67		
#69	depigmentation in od	
#70		
#71	nasal superior RD in od	Data not used
Intravitreal 1.5 μM (2hrs)		
#55		
#56		
#57	depigmentation in os	
#58	depigmentation in os	
Subretinal 0.5 μM (2 Days)		
#45A		
#45B	depigmentation in AC ou	
#47	cornea abrasion od	ERG data not used
#48	intraretinal hemorrhage in od (BSS)	
#49		
#50	depigmentation ou<br>Quantitative data were outliers.*	Data used only in Fig. 6.
#72	od (BSS).: retinal fold<br>os (AR13503).: bleeding occurred from sclerotomy, which resulted in coagulated blood in the vitreous; retina was still detached	Data not used
#74	depigmentation ou	
Subretinal 25 μM (2 Days)		
#51	Originally bigger RD (50 % of the retina)<br>10% of the retina was still detached ou.by 2 days.	Data not used
#52	Originally bigger RD (50 % of the retina)	Data used only in Fig. 6.
#53	os (BSS) had vitreous hemorrhage	Data used only in Fig. 6.
#54	depigmentation in AC in os	Data used only in Fig. 6.
#59	od (AR13503) still detached, os (BSS).: retinal fold,<br>ou: cloudy media	Data not used
#60	os (AR13503): PVR Grade C (Retina Society Classification 1991)	Data not used

Suppl. Table 1.: All animals tested during this study. Although pigs with dark, pigmented irides were requested for this study, to minimize the possibility of depigmentation in the posterior segment, some areas of depigmentation were present in the fundus of 15 eyes before surgery as noted. Reasons for the exclusion of some animals from data analysis are listed. *Retraction numbers after drug treatment were 2X higher than the average level of drug-treated eyes. (RD: retinal detachment; AC: area centralis; os: oculus sinister; od: oculus dexter; ou: oculus uterque; BSS: balanced salt solution; PVR: proliferative vitreoretinopathy)
